# PD-YOLO: A study of daily behavioural detection in housed sheep

**DOI:** 10.1371/journal.pone.0313412

**Published:** 2024-11-07

**Authors:** Jie Wang, Yahong Zhai, Lan Zhu, Longyan Xu, Hongguang Yuan

**Affiliations:** 1 School of Electrical and Information Engineering, Hubei University of Automotive Technology, Shiyan, China; 2 Qinyang Beisheng Pastoral Industry Co., Ltd., Qinyang, China; National University of Sciences and Technology NUST, PAKISTAN

## Abstract

Sheep behavior recognition helps to monitor the health status of sheep and prevent the outbreak of infectious diseases. Aiming at the problems of low detection accuracy and slow speed due to the crowding of sheep in real farming scenarios, which can easily obscure each other, this study proposes a lightweight sheep behavior recognition model based on the YOLOv8n model. First, the Convolutional Block Attention Module (CBAM) is introduced and improved in the YOLOv8n model, and the channel attention module and spatial attention module are changed from serial to parallel to construct a novel attention mechanism, PCBAM, to enhance the network’s attention to the sheep and eliminate redundant background information; second, the ordinary convolution in the backbone network is replaced with depth-separable convolution, which effectively reduces the number of parameters in the model and reduces the computational complexity. The study takes the housed breeding sheep as the test object, installs a camera diagonally above the sheep pen to collect images and makes a data set for testing, and in order to verify the superiority of the PD-YOLO model, compares it with a variety of target detection models. The experimental results show that the mean average precision (mAP) of the model proposed in this paper are 95.8%, 98.9%, and 96.2% for the three postures of sheep lying, feeding, and standing, respectively, which are 8.5%, 0.8%, and 0.8% higher than those of YOLOv8n, respectively, and the size of the model has been reduced by 13.3% and the amount of computation has been reduced by 12.1%. The inference speed reaches 52.1 FPS per second, which is better than other models in meeting the real-time detection requirement. To verify the practicality of this research method, the PD-YOLO model was deployed on the RK3399Pro development board for testing, and a high inference speed was achieved. It can provide effective technical support for sheep smart farming.

## Introduction

Sheep farming is an important agricultural activity worldwide and China, with its long history of sheep farming and abundant breeds, is one of the world’s leading countries in terms of sheep numbers [1]. As sheep production methods have changed, confinement has had certain effects on sheep growth. Compared to pasture farming, the higher stocking densities in large facilities limit the sheep’s range of movement and reduce their exercise levels. This can lead to a decline in the sheep’s immunity, which can trigger various diseases [2]. Healthy sheep are characterized by a strong appetite, frequent activity, and a tendency to feed first when food is available. Before the onset of the disease, there are often some abnormal behaviors, such as not feeding properly or standing in a corner. Therefore, the daily behavior of sheep can reflect their health status to a certain extent, and real-time monitoring of the behavior of the flock helps to timely judge the health status of the flock, detect and deal with potential problems in advance [3].

Traditional methods of individual behavior recognition for livestock mainly include installing accelerometer [4], gyroscope [5] and magnetometer [6] on the body. These sensors can help record the movement and posture of livestock, thereby helping to identify specific behaviors such as lying down, walking, or running [7]. Debeshi et al. [8] hung a multi-sensor Internet of Things device around a cow’s neck and analysed the data from the sensors using a random forest classifier to classify the cow’s actions, achieving a classification accuracy rate of 97%. Cornou et al. [9] fixed a triaxial accelerometer and Blue-tooth module on the sow’s neck, collected motion information, and wirelessly transmitted it to a computer. He used a multi-process Kalman filtering method to classify the sow’s behavior and predict the time of the sow’s delivery. Alvarenga et al. [10] quantitatively analysed the different voltage variation curves of the jaw or temporal fossa movements during sheep feeding and rumination using piezoelectric sensors and pressure strain gauges, enabling automatic recording of feeding and rumination data and other grazing behaviour.Turne et al. [11] classified sheep behavior under grazing conditions by attaching sensors to the mandibles and ears of grazing sheep to capture two sets of data.Although sensor technology provides many advantages in detecting livestock behavior, there are also some shortcomings. The data generated by sensors is usually a large amount of raw information, which may be difficult to interpret for non-professionals; sheep may cause wear or damage to their sensor devices during daily activities [12].

In recent years, the application of low-cost, non-contact image or video technologies in the agriculture and animal husbandry sector has been increasingly widespread [13–16].With the rapid development of deep learning in the field of computer vision, a large number of studies on livestock behavior recognition based on deep learning and computer vision have emerged [17].Yu et al. [18] proposed a method of ewe estrus recognition based on multi-object detection layer neural network. By adding object detection layer, introducing residual unit and optimizing data loading module, the recognition accuracy and model efficiency of ewe estrus behavior are significantly improved. Song et al. [19] proposed a sheep face detection method, which improves the recognition accuracy while reducing the model size by clustering anchor frames and compressing models, achieving low memory requirements, high recognition accuracy, and fast recognition speed. Zhang et al. [20] directly detects the drinking, urination, and climbing behaviors of sows by optimizing the deep learning network structure, which significantly improves the accuracy and real-time performance of behavior detection and meets the daily monitoring needs of most pig farms. Gu et al. [21] improved the accuracy of sheep behavior recognition through a two-stage method. In the detection stage, the improved network structure was used to achieve high-precision behavior classification, and in the classification stage, the VGG network was used to subdivide specific behaviors, which achieved good results. Fuentes et al. [22] proposed a cattle behavior recognition method based on deep learning that combines spatiotemporal information to detect and locate in video frames. Experimental results show that the system can effectively identify 15 different types of individual and group activities and partial actions. Liu et al. [23] extracted spatiotemporal features and classified behaviors by combining convolutional neural networks and recurrent neural networks, and overall was able to identify and locate 89.23% of the tail biting behaviors of herd pigs. Jiang et al. [24] proposed a general behavior recognition framework for herd-raised goats, and identified four goat behaviors by analyzing the spatial positional relationship between the goat bounding box and the feed and drinking areas, as well as the amount of movement of the center point of the same goat bounding box in consecutive frames.

However, research on typical motor behavior recognition in the process of raising sheep in captivity is not yet in-depth enough. With the transformation of breeding methods, behavior recognition of sheep in captivity has become increasingly urgent and necessary. Compared to other domestic animals, sheep exhibit stronger herd behavior. In limited confined feeding spaces, there is a serious occlusion problem during group gathering, and general detection algorithms are difficult to accurately detect and recognize sheep behavior. The main behaviors of sheep in narrow spaces include standing, eating, and lying down, which most intuitively reflect the health status of the sheep. To address these issues, this paper proposes an improved YOLOv8n-based behavior recognition algorithm for captive sheep, aiming to provide precise support for the intelligent breeding of sheep.

## Materials and methods

### Ethical statement

The research ethics review committee of Hubei Institute of Automotive Industry approved this study (2024LLSC04) and all methods were conducted in accordance with Hubei Institute of Automotive Industry Research Ethics Policy and the ethical guidelines of ISAE [25]. This study is only an animal behavior recognition study, does not involve animals themselves, and forcibly interferes with animal behavior. In this study, the environment and conditions of the sheep participating in the experiment before and after the study were consistent with those of other sheep in the farm. In our study, no invasive devices that interfered with the normal state of farm animals were used. We only recorded the video data with a camera. Before and after the data collection, the life of sheep on the farm did not change.

### Data sources

**Fig 1:**
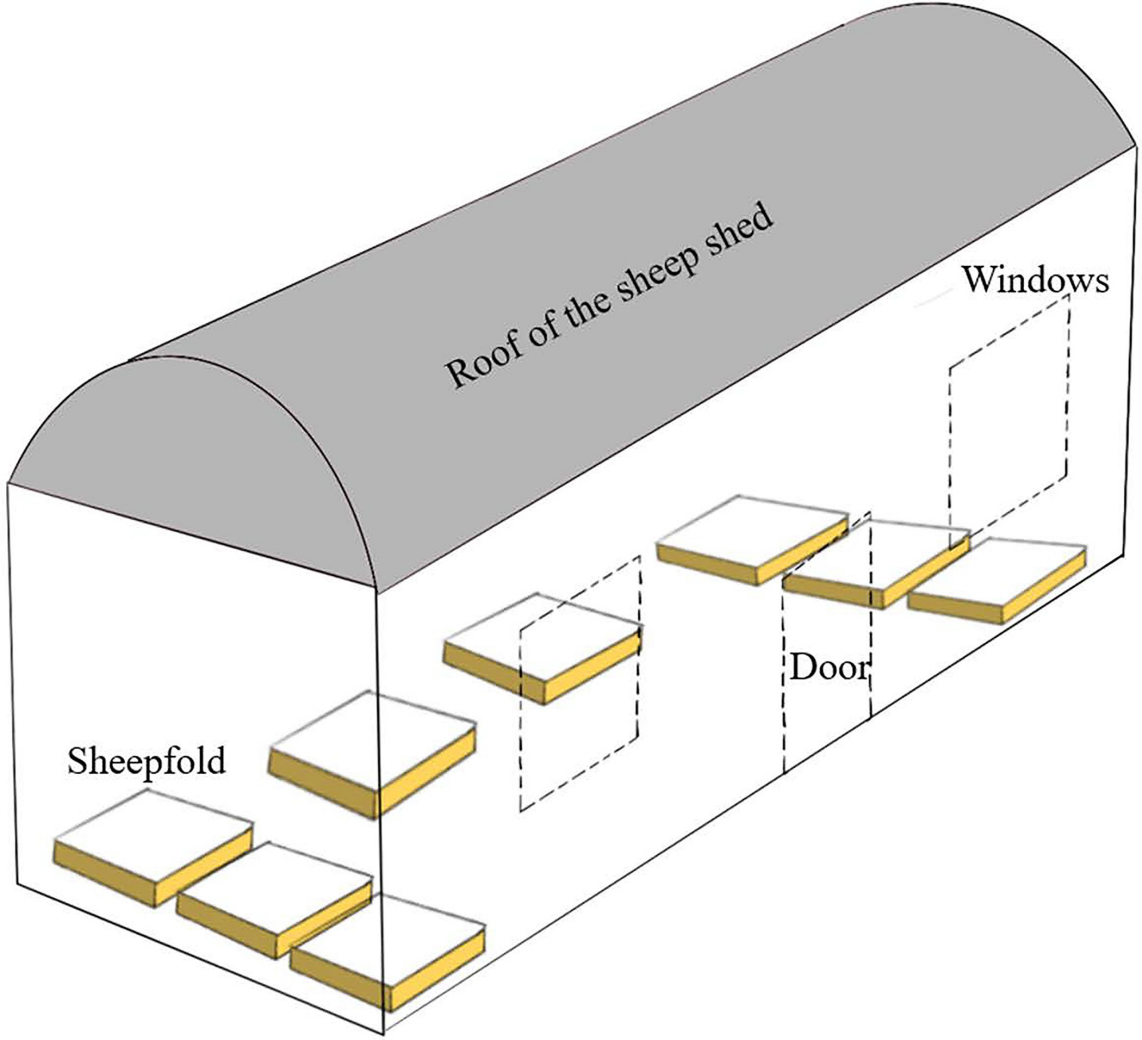
Schematic diagram of sheep house.

**Fig 2:**
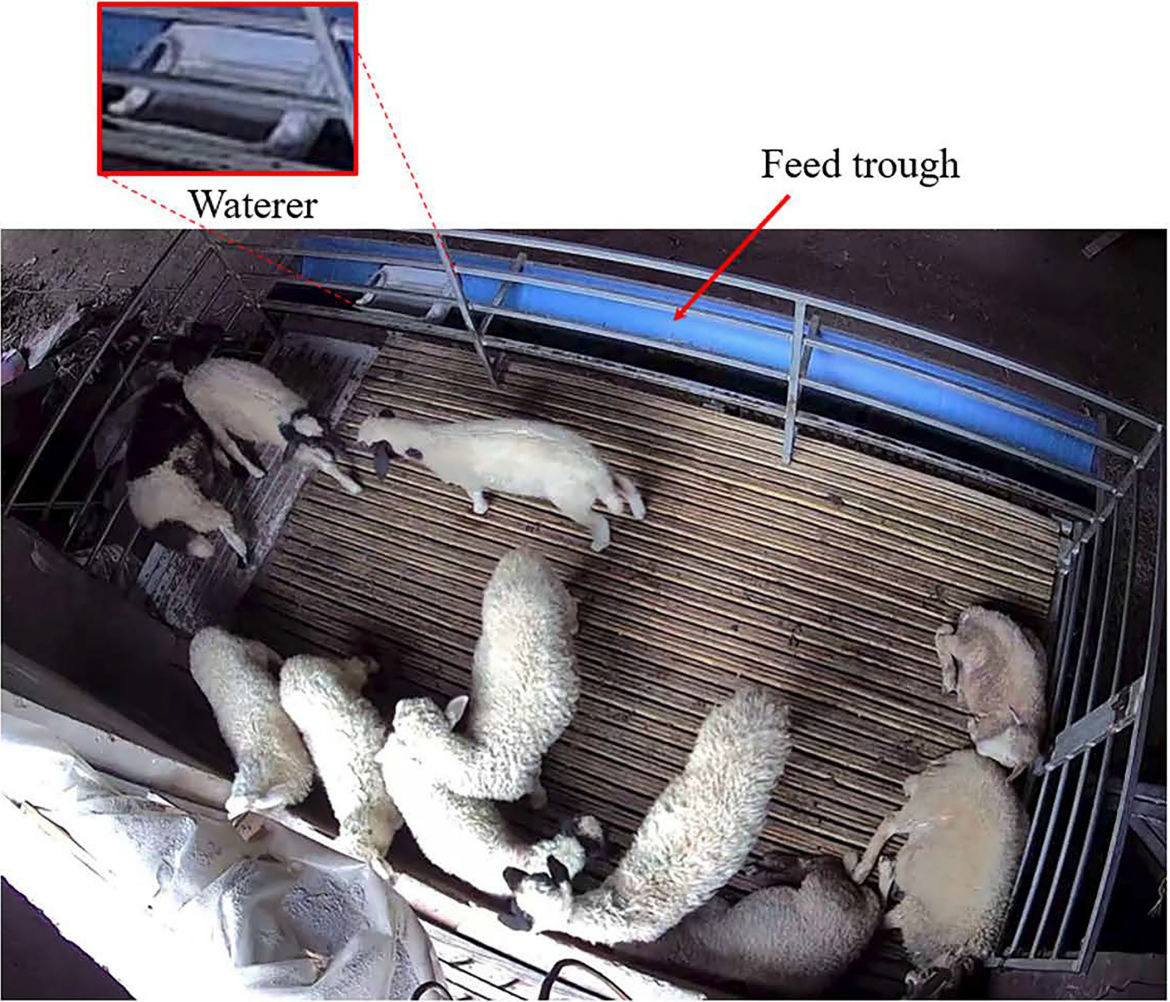
Layout of the sheepfold.

**Fig 3:**
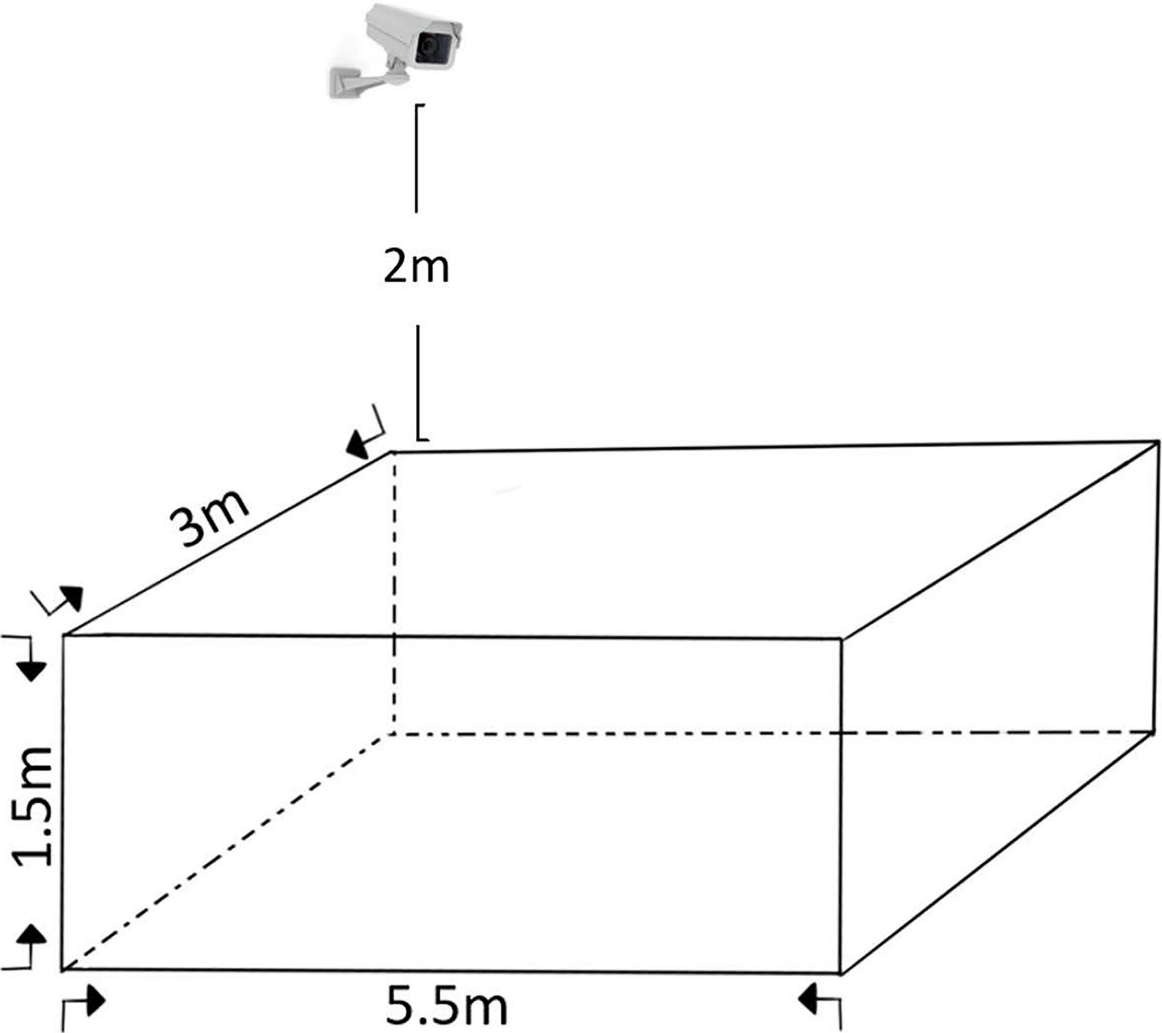
Camera Installation Schematic.

The sheep imagery was collected at the sheep breeding farm of Beisheng Pastoral Industry Co., Ltd., located in Qinyang City, Henan Province. For the purposes of this study, a semi-open sheep shed was employed, with each shed accommodating nine sheep pens, as depicted in Fig 1. Each pen was equipped with a water dispenser and a feed trough, as illustrated in Fig 2. The data collection period spanned from July to August 2023. Subjects for image collection were chosen from a pen that housed a dense population of mixed-sized breeds of sheep. To address potential variations in lighting conditions, the camera was mounted diagonally above the window of the sheep pen. An image acquisition system was established to record top-down videos of the sheep, capturing their activities on video. The camera model is the Xiaomi CW500, with 5 megapixels and an f/1.6 wide aperture lens. The schematic diagram of the camera’s placement is illustrated in Fig 3.

### Data preprocessing

**Fig 4:**
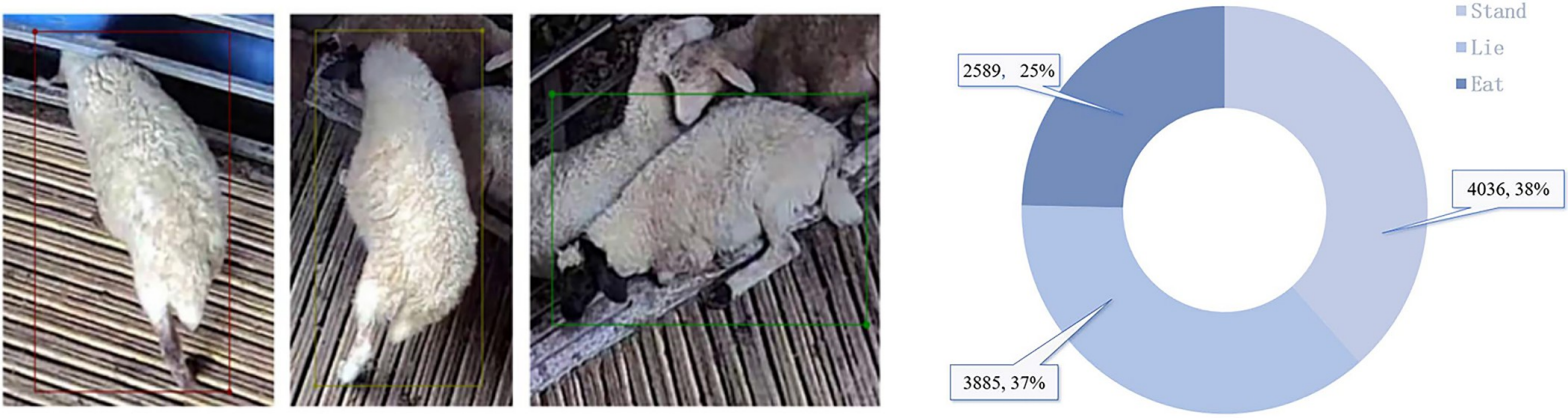
The red rectangle indicates sheep feeding behavior, the yellow rectangle indicates sheep standing behavior, and the green rectangle indicates sheep lying behavior. Number and proportion of different behaviors in the daily behavior data set of housed sheep.

After a preliminary manual screening, 300 videos of more frequent sheep activity were retained, each video being 2 seconds in length. One frame per second was captured from the video using video frame splitting techniques, resulting in a total of 1200 images. The images were re-screened to remove motion blur and retain 1185 usable images. The images were labeled with the three behaviors of feeding, lying and standing using the Labelme tool. The different sheep behaviors and the number of behaviors labeled are shown in Fig 4. The labeled images are divided into training set, validation set and test set according to 6:2:2.

**Fig 5:**
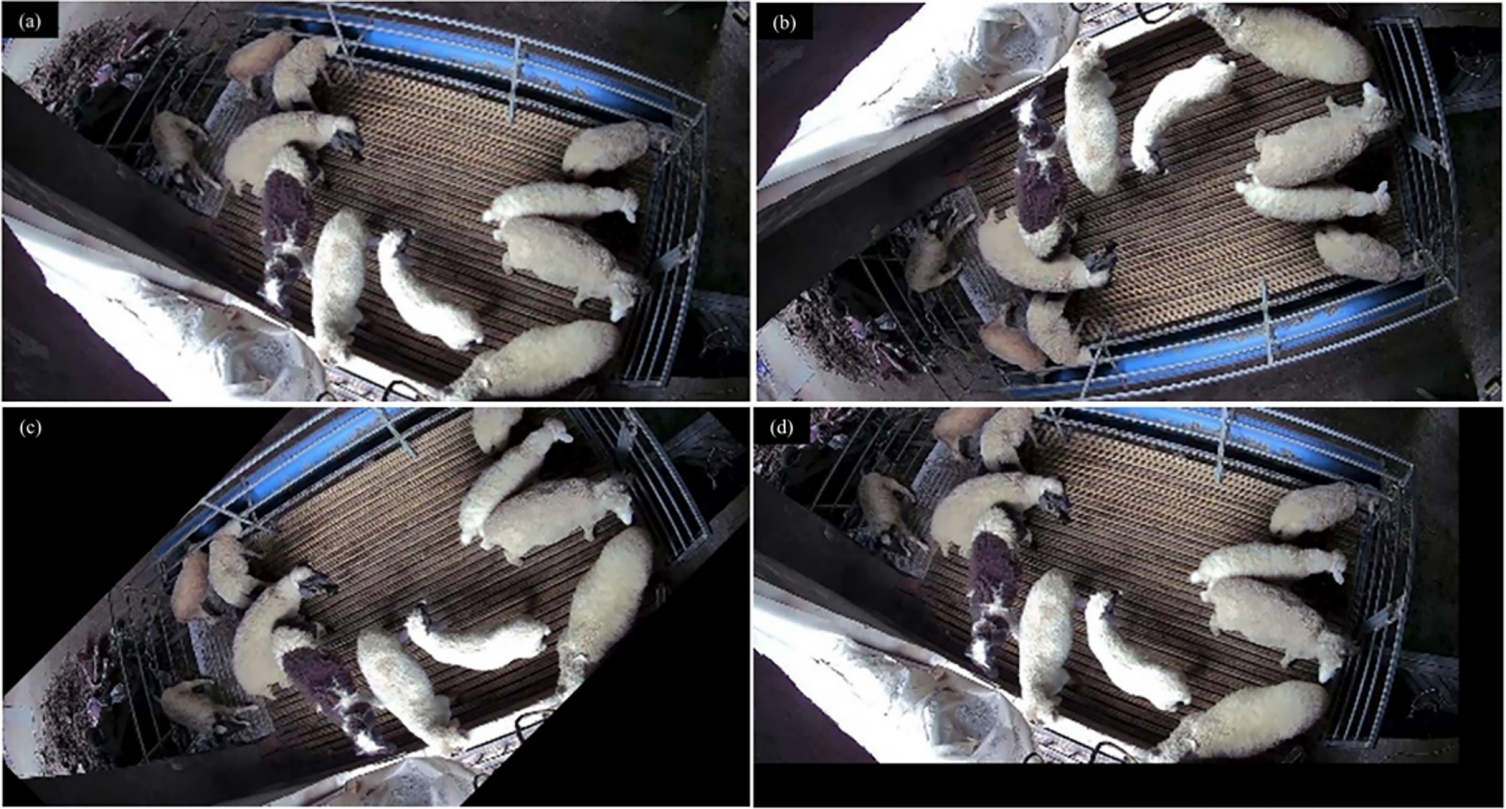
Data Augmentation. (a) Raw image; (b) Transposed image; (c) Rotated image; (d) Translated image.

In order to improve the robustness and generalization ability of the network model, data enhancement methods such as panning, up and down flipping are used to expand the diversity of data in the training set, and the training set is expanded three times after data enhancement. The effect of the data enhancement process is shown in Fig 5.

### PD-YOLO model

YOLOv10 is the latest model of YOLO series target detection model [25–29]. Based on YOLOv8, it has improved the lightweight classification head and the undersampling layer. is helpful to improve the detection speed, but is weaker than YOLOv8 in the detection of complex scenes.According to the different network depth and width, the YOLOv8 model is divided into five versions: YOLOv8x, YOLOv8l, YOLOv8m, YOLOv8s and YOLOv8n, to adapt to different application scenarios. The larger the computational and parametric sizes of the model, the higher the recognition accuracy, but the slower it is. Considering the computational speed and real-time performance, this paper adopts the YOLOv8n model as the bench-mark model for improvement.

The YOLOv8n model consists of four main parts: the input layer, the backbone net-work, the neck network, and the head network.Adaptive image scaling is used in the input layer to adjust the input size, and mosaic data augmentation is also used on the input images to introduce more variation and diversity, which makes the model more robust. The backbone network consists of the CBL module, the C2f module, and the SPPF module.The CBL module encapsulates the convolution, batch normalization, and activation function, which improves the stability of the model.The C2f module fuses the ELAH structure of the CSPNet [30] and the YOLOv7, which achieves lightweighting and improves inference.The SPPF module, through the average pooling and maximum pooling operations, is able to adaptively fuse features of different scales to improve the feature ex-traction capability of the model. The neck network uses a Feature Pyramid Network [31] and a Path Aggregation Network [32] to improve model performance by allowing features extracted from the backbone network to be more fully fused at all levels through top-down and bottom-up cross-layer connections. YOLOv8n replaces the detection header with the current mainstream decoupling header, which separately extracts the target location and category information, and learns them through different network branches are learned separately and then fused, which effectively reduces the number of parameters and computational complexity, and improves the generalization ability and robustness of the model.

**Fig 6:**
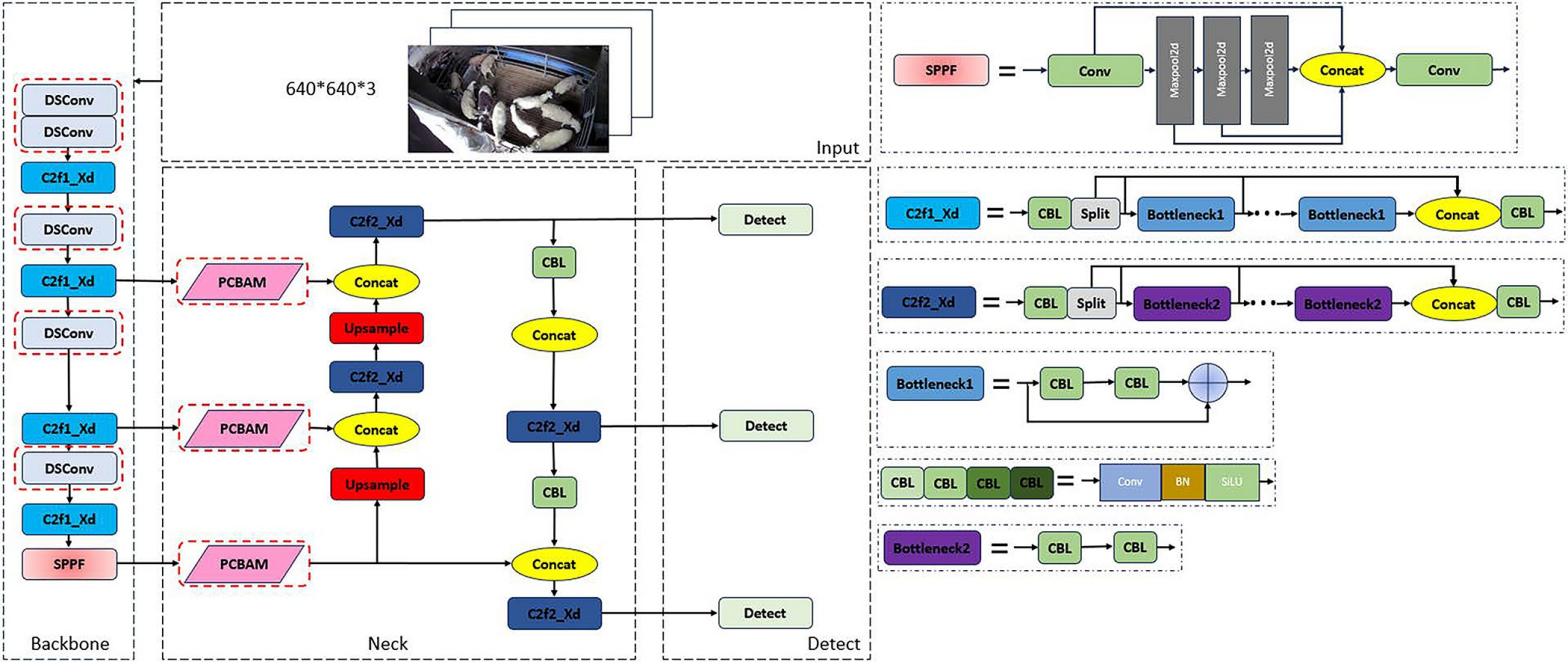
The architecture of the proposed PD-YOLO.

Sheep tend to flock together and are prone to mutual occlusion, and the YOLOv8n model suffers from insufficient robustness in detecting heavily occluded targets. In addition, existing algorithms are often difficult to deploy on resource-constrained hard- ware platforms while balancing real-time and accuracy requirements. Aiming at the above problems, this paper improves the YOLOv8n model; by improving the introduced CBAM module [33], the weights of each position in the feature map are dynamically adjusted according to the contextual information, focusing on the key features when detecting the occluded targets, and improving the model’s ability of detecting the oc cluded targets; and by using the depth-separable convolution DSConv [34] instead of the ordinary convolution in the trunk network, thus lightweighting the network. The ordinary convolution is used to lighten the network structure and reduce the number of model parameters. It is worth noting that we adopt the default loss function of the YOLOv8 model, which consists of three main parts: Location Loss, Classification Loss, and Objectness Loss. The specific implementation of these loss functions follows the settings of the original YOLOv8 model without any modification. The improved network model structure is shown in Fig 6.

### PCBAM model

**Fig 7:**
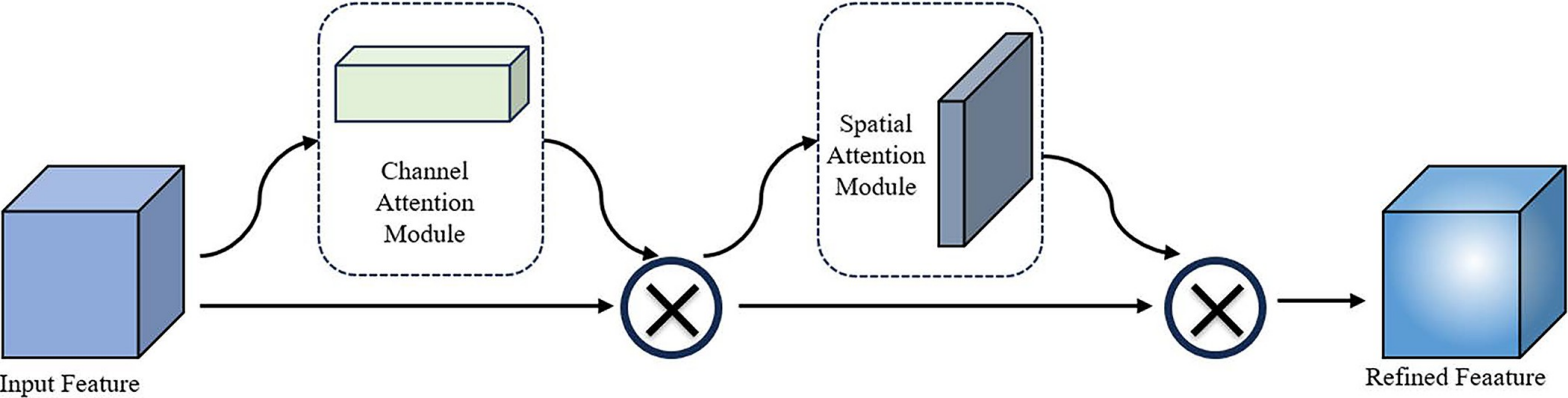
Convolutional Block Attention Module structure.

The Attention Mechanism is a method that attempts to emulate the human visual and cognitive systems. In image recognition tasks, it effectively reduces the interference caused by background noise, allowing neural networks to focus more on the salient feature regions of the target object. Consequently, it has been widely applied. The CBAM module is a lightweight universal attention mechanism module that is primarily employed to enhance the attention of Convolutional Neural Networks to different feature channels and spatial positions, thereby enhancing the model’s ability to extract complex features. The CBAM module structure is depicted in Fig 7. If the input feature map is represented by the following equation: *F*∈*R*^*C*×*H*×*W*^, where *F* is the input feature map, *H* is height, *W* is width, and *C* is the number of channels. The CBAM module employs a two-step process to infer the attention map. First, it considers the channel dimension and spatial dimension, and then it multiplies the attention map with the input feature map to adaptively refine the features. The mathematical expression of this is as follows:

$$F^{\prime }=M_{C}(F)⊗F$$ (1)

$$F^{\prime \prime }=M_{S}(F^{\prime })⊗F^{\prime }$$ (2)

where *⊗* represents element-wise multiplication, *M*_*C*_(*F*) represents the output weights of the input feature map after passing through channel attention, and *M*_*S*_(*F*’) represents the output weights of spatial attention.

**Fig 8:**
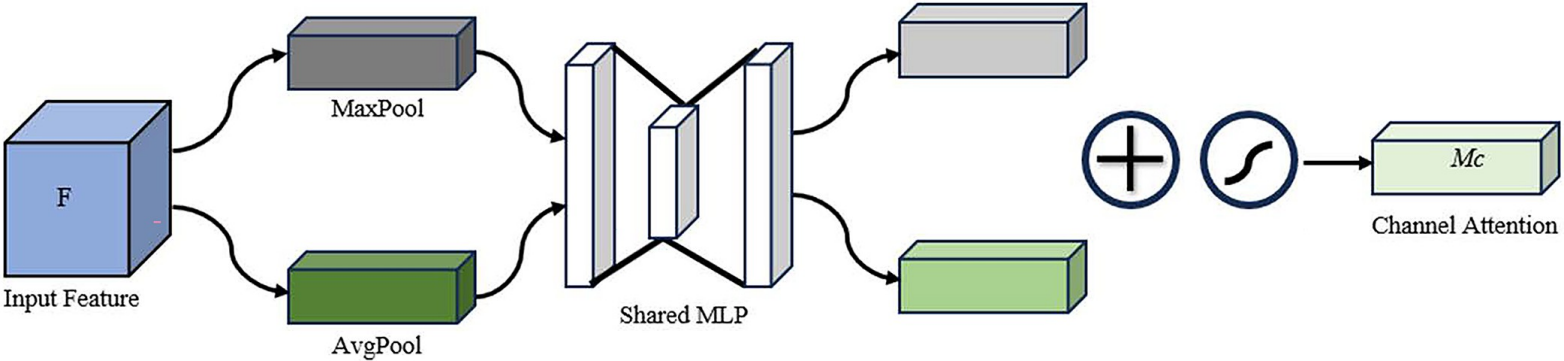
Channel attention module.

**Fig 9:**
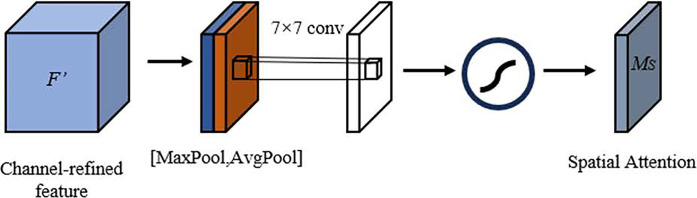
Spatial attention module.

In contrast to the conventional single-channel and spatial attention mechanisms, the CBAM attention mechanism integrates the Channel Attention Module and the Spatial Attention Module in a serial manner, thereby combining these two sub-modules. The CBAM module infers attention maps along two separate dimensions in sequence, and then multiplies the attention maps with the input feature map for adaptive feature optimisation. This process is initiated by the module when an intermediate feature map is provided. The sub-modules of the CBAM module are shown in Figs 8 and 9 respectively.

In the diagram, the input feature map is subjected to max pooling and average pooling operations, respectively, in order to obtain the max pooling vector and the spatial pooling vector. Following their passage through a shared fully connected layer, two vectors of the same dimension are obtained. Subsequently, the aforementioned vectors are added together and passed through a sigmoid activation function, thereby yielding the channel attention weights *M*_*C*_. The feature map that has undergone channel attention processing is then input into the spatial attention module, where it undergoes max pooling and average pooling operations, resulting in two 1 *HW* feature maps. The aforementioned feature maps are then concatenated to form a single 2 *HW* feature map. Subsequently, the aforementioned feature map is subjected to a convolutional layer with a kernel size of 7×7, after which it is passed through a sigmoid function. This process yields the spatial attention weights, *M*_*S*_. The calculation process for *M*_*C*_ and *M*_*S*_ is as follows:

$$\begin{array}{cc} M_{C}(F) & =\sigma (MLP(AvgPool(F))+MLP(MaxPool(F)))\\ & =(W_{1}(W_{0}(F_{avg}^{C}))+W_{1}(W_{0}(F_{max}^{C}))) \end{array}$$ (3)

$$M_{S}(F)=\sigma (f^{7\times 7}([AvgPool(F);MaxPool(F)]))=\sigma (f^{7\times 7}([F_{avg}^{S};F_{\max}^{S}]))$$ (4)

where *M*_*C*_(*F*) represents the channel attention weights; MaxPool refers to global max pooling; AvgPool refers to global average pooling; MLP denotes the shared fully connected layer;*W*_1_ and *W*_0_ are the weights of the fully connected layer; $F_{\mathrm{avg}}^{C}$ and $F_{\max}^{C}$ are the average pooling vector and the max pooling vector, respectively; *M*_*S*_ represents the spatial attention weights; *f*^7×7^ denotes the convolution operation with a kernel size of 7×7.

**Fig 10:**
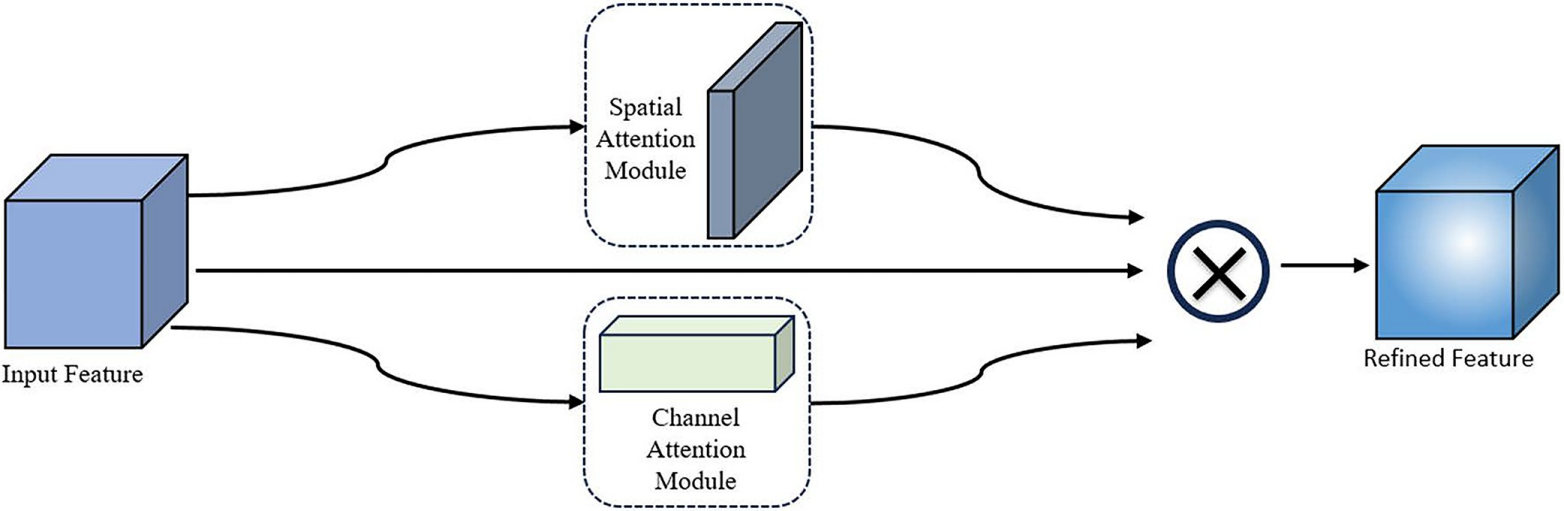
PCBAM structure.

The CBAM module has good performance in computer vision tasks, but has some limitations in the specific task of behavioral recognition of housed sheep. Although channel attention followed by spatial attention can gradually refine the feature map, it may limit the features learned by spatial attention. Channel attention in parallel with spatial attention may be more efficient in capturing the features of individual sheep and locating where the behavior occurs. Therefore, this study improves CBAM by changing the connection of the two attention modules from”serial” to”parallel”, i.e., PCBAM, and the overall structure is shown in Fig 10.

The corresponding weights are obtained from the input feature map *F* by spatial and channel attention, respectively, and then the output weights are directly weighted with the original input features to obtain the output feature map *F*’. The calculation formula is as follows:

$$F^{\prime }=M_{S}(F)⊗M_{C}(F)⊗F$$ (5)

### Depthwise separable convolution

Although YOLOv8n is a small model, it still requires a certain amount of computational resources for real-time target detection, which is still difficult to implement in practice for edge devices with weak computational power, so it is necessary to reduce the number of references and reduce the model size.

**Fig 11:**
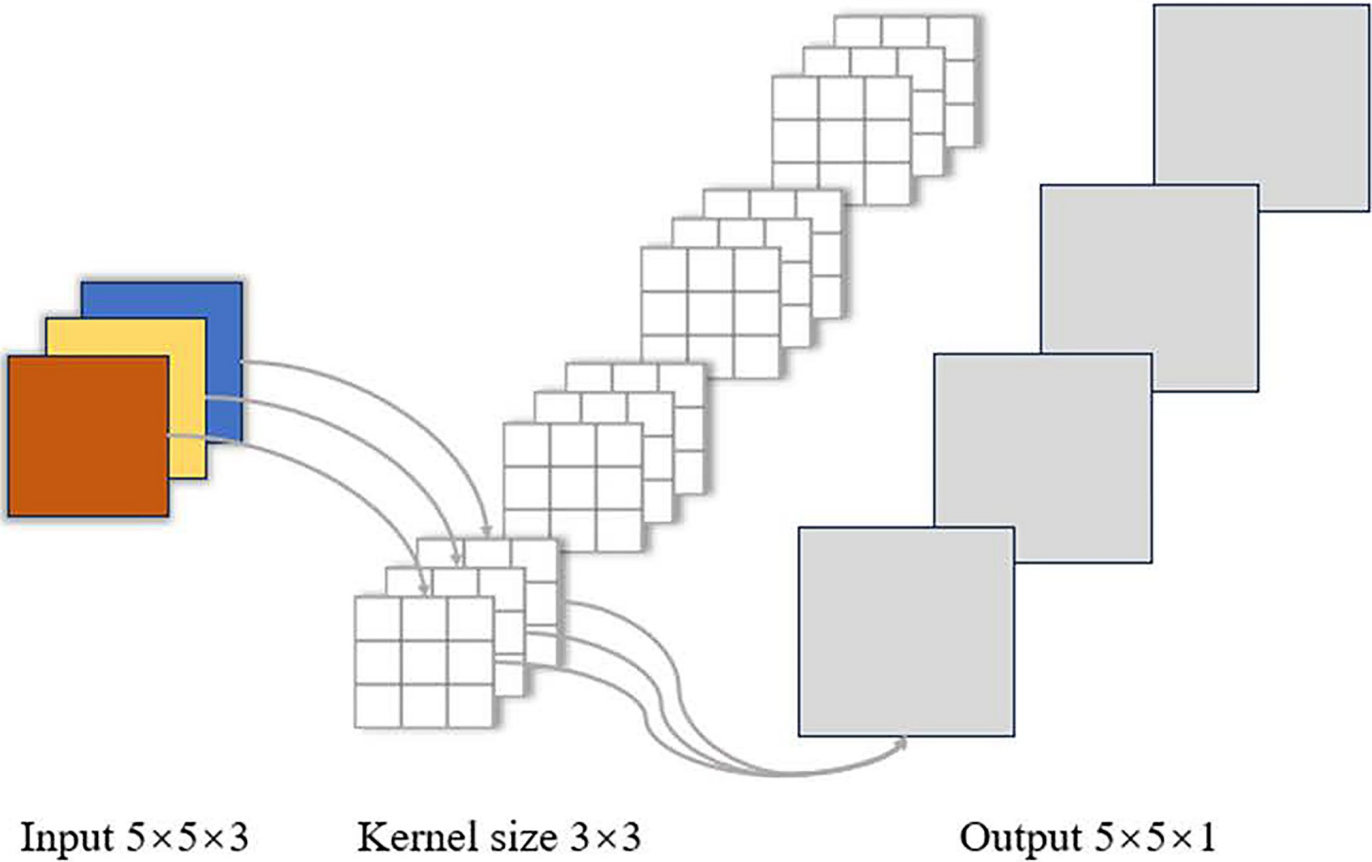
Ordinary convolution module structure.

Convolution is an indispensable component of neural network models, and is used in convolutional neural networks to extract image features and input these features to the classification layer for image classification. Ordinary convolution is the extraction of local information within each channel by the convolution kernel and the integration of information from all channels to obtain the final convolution output, with the char- acteristics of weight distribution and local connectivity, which can effectively extract the features in the image, but the number of its parameters is relatively large, and the computational cost is high. Taking a 5×5×3 image as an example, the structure of the ordinary convolution module is shown in Fig 11.

**Fig 12:**
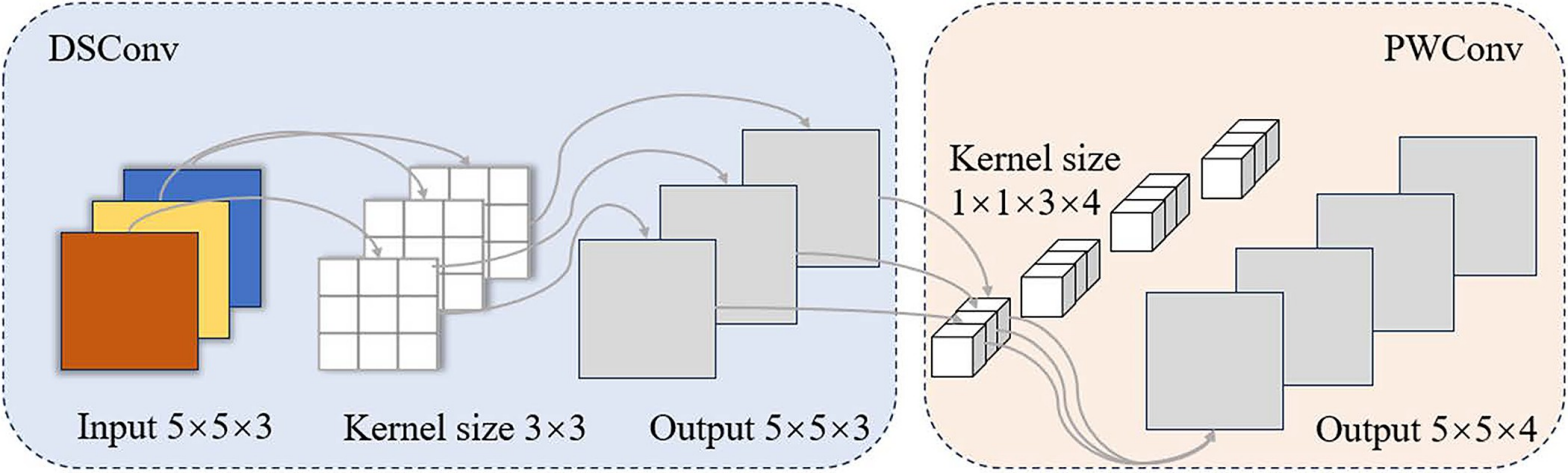
DSConv module structure.

Depth separable convolution consists of two parts, Depthwise Convolution (DWConv) and Pointwise Convolution (PWConv). Separating channel convolution and spatial convolution, channel convolution corresponds to DWConv and spatial convolution corre- sponds to PWConv. Unlike ordinary convolution, in deep convolution, each convolution kernel is responsible for convolution of only one channel, so each convolution kernel has only one dimension, the number of convolution kernels is equal to the number of channels of the input, and the number of channels of the output is also equal to the number of channels of the input. Point-by-point convolution is similar to ordinary convo- lution in that it generates a new feature map of the same size as the input by weighted combination in the direction of channel depths, and achieves dimensional transformation and inter-channel information interaction with less computation, thus improving model performance. In this study, DSConv is used instead of ordinary convolution. Compared with ordinary convolution, DSConv has almost the same feature extraction capability as ordinary convolution, while being more lightweight. Taking a 5×5×3 image as an example, the structure of the DSConv module is shown in Fig 12.

If the number of channels of the input feature map is *S*, the size of the convolution kernel is *M*, and the number of convolution kernels is *N*, the formulas for the number of parameters *A*_1_ for ordinary convolution and *A*_2_ for DSConv convolution are given in Eqs 6 and 7, respectively:

$$A_{1}=M\times M\times S\times N$$ (6)

$$A_{2}=M\times M\times S\times +S\times N$$ (7)

Therefore, the number of parameters of DSConv is only Δ_*A*_ of the normal convolution, as shown in Eq 8:

$$△A=\frac{A_{2}}{A_{1}}=\frac{1}{N}+\frac{1}{M^{2}}$$ (8)

## Results and analysis

### Experiment environment

The operating system version used in this experiment is Ubuntu 18.04.5 LTS, the CPU is Intel Xeon processor, the GPU is NVIDIA GeForce RTX 3060, the CUDA version is 11.7, the deep learning framework is Pytorch 1.13.1, and the compilation environment is Python 3.7.Model The detailed parameters for training are shown in Table 1.

**Table 1:** Model training parameters.

Hyperparameters	Value
*ImageSize*	*640×640*
*Epoch*	*200*
*Optimization*	*SGD*
*Batchsize*	*16*
*Learingrate*	*0*.*01*

### Evaluation metrics

A total of six evaluation metrics are employed in order to provide a comprehensive assessment of the detection model. These include precision, recall, parameter count, floating-point computations(FLOPs) [35], mean average precision (mAP), and inference speed in frames per second (FPS). The equations for these metrics are as follows:

$$P=\frac{TP}{TP+FP}$$ (9)

$$R=\frac{TP}{TP+FN}$$ (10)

$$AP=\int_{0}^{1}P(R)dR$$ (11)

$$mAP=\frac{\sum_{0}^{1}AP_{1}}{n}$$ (12)

TP represents the number of positive samples correctly predicted as positive by the model, FP represents the number of negative samples incorrectly predicted as positive by the model, and FN represents the number of positive samples incorrectly predicted as negative by the model. AP represents the area under the Precision-Recall (P-R) curve, while mAP denotes the average of AP for each category.

### Comparison experiment of attention mechanism modules

To verify the effectiveness of our proposed PCBAM module, this experiment compares the CBAM module with the PCBAM module and some common attention mechanism modules [36–38], and the experimental results are shown in Table 2.

**Table 2:** Comparison of attention mechanisms.

Model	Parameters(MB)	FLOPs (G)	mAP50 (%)
*YOLOv8n*	*3*.*0*	*8*.*1*	*93*.*6*
*YOLOv8n−E*	*3*.*3*	*9*.*3*	*94*.*5*
*YOLOv8n−EC*	*3*.*2*	*9*.*0*	*93*.*7*
*YOLOv8n−S*	*3*.*0*	*8*.*1*	*90*.*5*
*YOLOv8n−C*	*3*.*0*	*8*.*1*	*95*.*3*
*YOLOv8n−P*	*3*.*0*	*8*.*1*	*96*.*6*

YOLOv8n-E represents the addition of the SE attention module; YOLOv8n-EC represents the addition of the ECA attention module; YOLOv8n-S represents the addition of the SimAM attention module; YOLOv8n-C represents the addition of the CBAM attention module; YOLOv8n-P represents the addition of the PCBAM attention module.

From the experimental results, it can be seen that most of the models with the added attention module have higher mean average precision than YOLOv8n, and only the mean average precision of the YOLOv8n-S model is lower than the benchmark model. This is probably due to the fact that SimAM’s attention mechanism only considers attention in the spatial dimension, and is unable to capture attention in the channel dimension, which is easily affected when dealing with images with occlusion; The YOLOv8n-E model has a mean average precision of 94.5%. The YOLOv8n-P model has the best performance among all models, with a mean average precision of 96.6%, which is a 3% improvement over YOLOv8n, and there is no significant increase in the number of parameters and the amount of computation. The YOLOv8n-C model was the next best with a mean average precision of 95.3%.

### Visualization analysis of attention mechanism

Heat maps of attentional mechanisms can visualize which regions of the graph the target detection model is more interested in and, to some extent, visualize the detection results. In this study, we use the Gradient-weighted Class Activation Mapping (Grad- CAM) method [39] to visualize the features from six sets of attention module ablation experiments.Grad-CAM is a gradient-based network visualization method that uses the gradient of the last convolutional layer to calculate the weight of each channel, and maps the weighted feature map onto the original image in the form of a heat map, where the pixel value represents the importance of that pixel region for the classification result.

**Fig 13:**
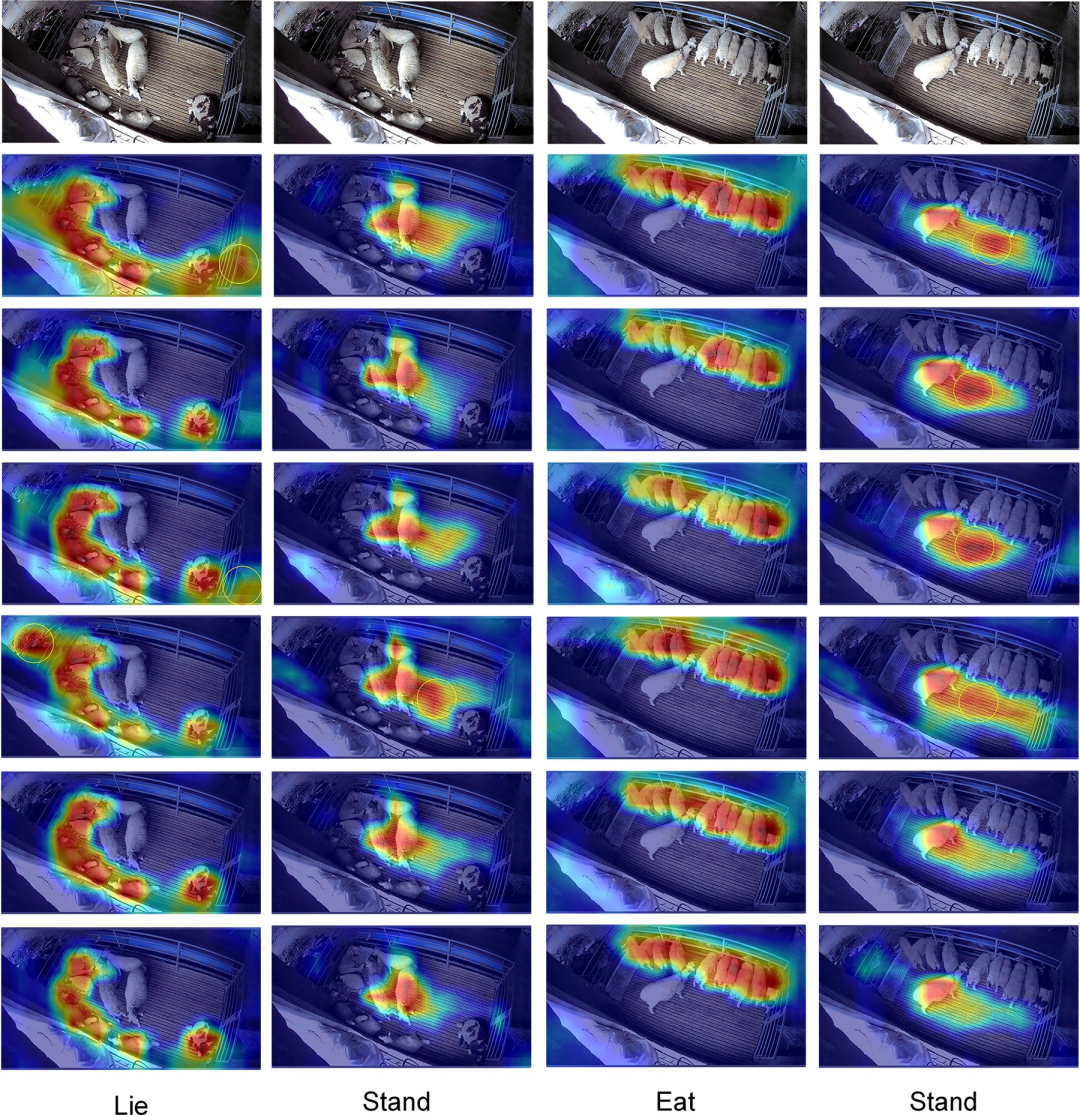
Heat map of attention mechanisms. The first row shows the raw image, the second row is YOLOv8n, the third row is YOLOv8n-E, the fourth row is YOLOv8n-EC,the fifth row is YOLOv8n-C, the sixth row is YOLOv8n-S, and the seventh row is YOLOv8n-P.

The visualization results are shown in Fig 13. It can be seen that the model detection heat map with the PCBAM attention module is closer to the real sheep region. In the area surrounded by the yellow circle, the YOLOv8n model, the YOLOv8n-E model, the YOLOv8n-EC model, the YOLOv8n-C model, and the YOLOv8n-S model all show strong thermal values even without the presence of sheep, which is prone to the false detection phenomenon. When detecting the standing behavior of sheep, the attention thermograms of the YOLOv8n-P model were slightly overflowed from the real situation, but still the best performance among all models. The visualization results show that the PCBAM module can extract stronger features from the target.

### Ablation experiment

In order to ascertain the extent to which different optimisation strategies enhance the performance of the YOLOv8n model, four sets of ablation experiments were conducted. The results of the ablation experiments are presented in Table 3.

**Table 3:** Results of ablation experiments.

PCBAM	DSConv	mAP50 (%)	Parameters(MB)	FLOPs (G)
		*93*.*6*	*3*.*0*	*8*.*1*
✓		*96*.*6*	*3*.*0*	*8*.*1*
	✓	*93*.*8*	*2*.*6*	*7*.*2*
✓	✓	*96*.*9*	*2*.*6*	*7*.*2*

‘✓’ indicates that a corresponding improvement has been made.

The mean average precision of YOLOv8n for sheep behavior detection is 93.6%; by incorporating the PCBAM module into the neck network to improve the model’s focus on sheep and eliminate redundant background information, the mean average precision is improved by 3% without significantly increasing the number of model parameters and computational effort; by replacing the ordinary convolution in the original model back-bone network with the DSConv convolution only, the number of model parameters and computational effort are reduced by 0.4M and 0.9G, respectively. Although the DSConv convolution simplifies the computational process, its decomposition retains the model’s ability to capture the local spatial features and cross-channel feature interactions, so the mean average precision is improved by a small amount; in the case of adding the PCBAM module and replacing the DSConv convolution at the same time, the model detection per-formance achieves the best performance, and the mean average precision is improved to 96.9%.

### Performance analysis of algorithms under varying degrees of occlusion

**Fig 14:**
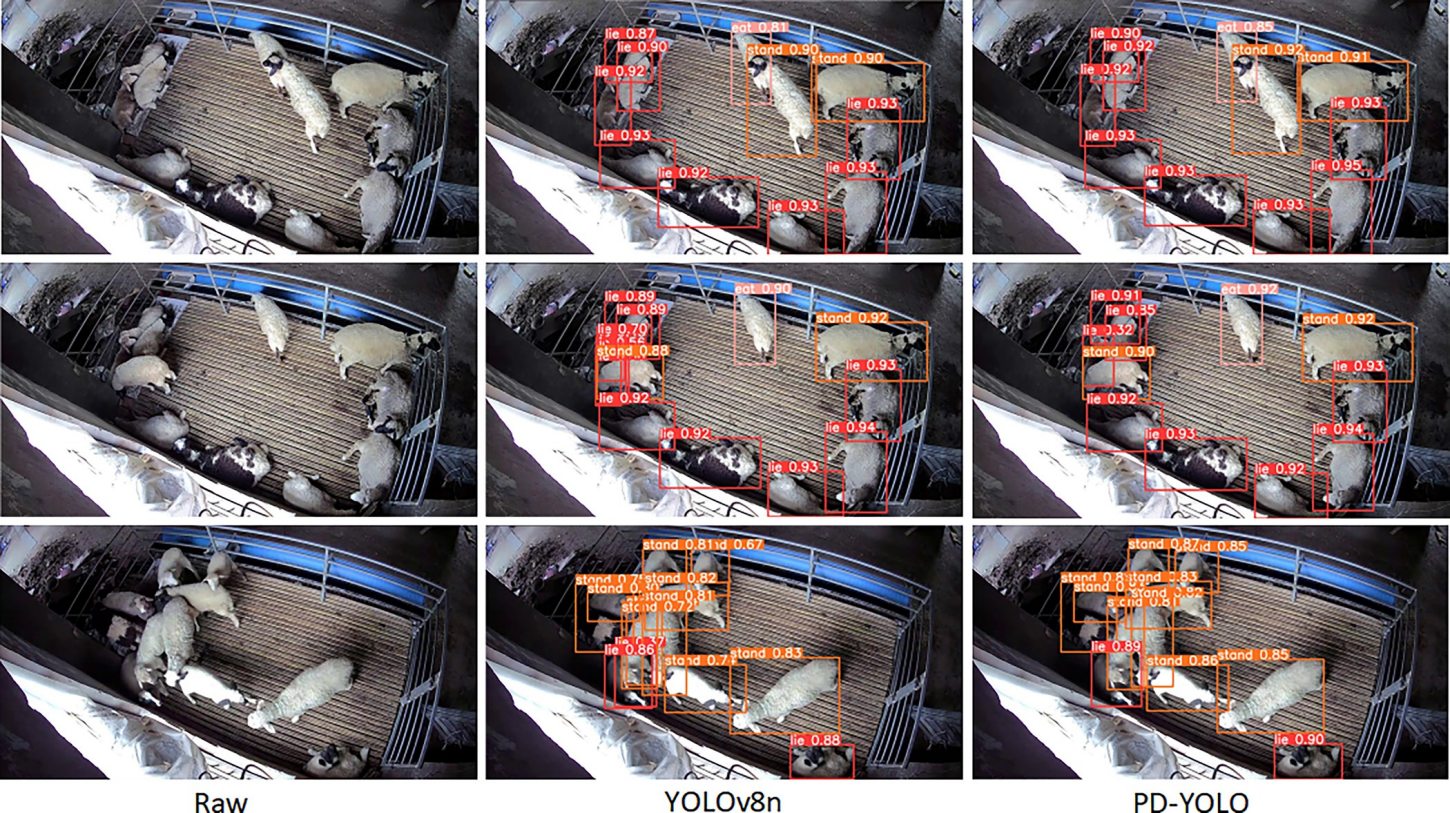
Sheep behavior detection in three occlusion scenarios. The first row shows lightly occluded images, the second row shows moderately occluded images, and the third row shows heavily occluded images.

In order to assess the efficacy of the proposed methodology for the detection of occluded sheep, a verification process was conducted under three distinct levels of occlusion: slight, moderate, and severe. The detection examples presented under the three aforementioned scenarios are illustrated in Fig 14.

It can be observed that the accuracy of the detection is often affected when sheep are crowded or occluded. The YOLOv8n model exhibited a decline in performance in the moderate and severe occlusion scenarios, attributed to the limited effective features ex-tracted, which resulted in an increase in missed detections and false detections. Neverthe-less, the decline in performance is not substantial in the case of slight occlusion. The pro-posed method demonstrates satisfactory performance in moderate and severe occlusion scenarios. In conclusion, the PD-YOLO network model demonstrates robust performance in occlusion scenarios, enabling accurate localisation and detection of sheep in a variety of occlusion conditions.

### Comparative analysis with other models

To further analyze and verify the effectiveness of the PD-YOLO model in the sheep behavior detection task, it is compared and experimented with representative one-stage target detection algorithms RTMDet [40], the YOLO series, and the two-stage target detection algorithm Faster R-CNN [41]. To ensure fairness, the experiments follow the following consistent conditions: (1) using the same performance evaluation index system; (2) ensuring the same training strategy; (3) training and reasoning under the same hardware environment (e.g., GPU model, memory size); and (4) all models are trained and tested on the sheep behavior dataset proposed in this paper. The experimental results are presented in Table 4.

**Table 4:** Comparison of different models.

Model	Backbone	mAP50 (%)			mAP50 (%)	Parameters(MB)	FLOPs (G)	FPS(Frame/s)
		Lie	Stand	Eat				
*FasterR−CNN*	*ResNet50*	*86*.*3*	*83*.*1*	*83*.*8*	*84*.*4*	*83*.*7*	*194*.*3*	*33*.*2*
*RTMDet*	*CSPNeXt*	*93*.*3*	*92*.*5*	*96*.*2*	*94*.*0*	*52*.*3*	*50*.*8*	*45*.*0*
*YOLOv3*	*ResNet50*	*91*.*3*	*83*.*5*	*89*.*2*	*88*.*0*	*61*.*7*	*155*.*3*	*22*.*4*
*YOLOv4s*	*CSPDarknet53*	*94*.*7*	*91*.*1*	*97*.*1*	*94*.*3*	*76*.*2*	*16*.*9*	*35*.*0*
*YOLOv5n*	*CSPDarknet53*	*90*.*1*	*89*.*5*	*95*.*2*	*91*.*6*	*1*.*77*	*4*.*7*	*35*.*8*
*YOLOv6n*	*RepVGG*	*88*.*0*	*84*.*5*	*90*.*9*	*87*.*8*	*4*.*5*	*11*.*4*	*23*.*6*
*YOLOv7−tiny*	*Darknet53*	*92*.*7*	*87*.*2*	*91*.*0*	*90*.*3*	*6*.*1*	*13*.*6*	*44*.*3*
*YOLOv8n*	*Darknet-53*	*93*.*0*	*92*.*4*	*95*.*4*	*93*.*6*	*3*.*0*	*8*.*2*	*48*.*9*
*YOLOv9−T*	*CSPDarknet-53*	*94*.*1*	*80*.*0*	*93*.*5*	*89*.*2*	*2*.*0*	*7*.*7*	*45*.*7*
*YOLOv10n*	*Darknet-53*	*87*.*9*	*85*.*6*	*92*.*0*	*88*.*5*	*2*.*7*	*8*.*4*	*50*.*9*
*PD−YOLO*	*Improve Darknet-53*	*97*.*4*	*94*.*5*	*98*.*8*	*96*.*9*	*2*.*6*	*7*.*2*	*52*.*1*

Table 4 shows the recognition results of the above nine models for the lying, feeding and standing behaviors of sheep. From Table 4, it can be seen that the Faster R-CNN model has low detection accuracy and the largest number of model parameters, with the largest computational volume; the YOLOv3 model ranks second, although the mean average precision is higher than that of the Faster R-CNN, but due to the large number of model network parameters, resulting in slow inference, which makes it difficult to meet the requirements of real-time detection; The mean average precision of YOLOv5n, YOLOv6n, YOLOv7- tiny,YOLOv9-T and YOLOv10n mean average precision is lower than that of YOLOv8n model, and the inference speed of YOLOv6n model does not meet the requirements of real-time detection; RTMDet model has a higher mean average precision, but the number of model parameters is higher, which can not meet the requirement of lightweight. The average accuracy mean of the standing pose is usually the lowest among all the models, probably because the standing pose is less distinguishable from other poses and is affected by more subtle movement changes. Compared with the above eight models, the PD-YOLO model proposed in this paper has the highest average accuracy in lying, eating, and standing behaviors, reaching 97.4%, 98.8%, and 94.5%, respectively, with an overall average accuracy of 96.9%,which is 12.5%, 2.9%, 8.9%, 2.6%, 5.3%, 9.1%, 6.3%, 3.3%, 7.7% and 8.4% higher than the other models, and in terms of model memory occupancy and computation, it is only slightly higher than the YOLOv5n and YOLOv9-T model, meeting the lightweight requirements, can be used on low computing power edge devices, inference speed reached 52.1FPS, is the highest of all models, compared with YOLOv8 improved 6.5%, to meet the requirements of real-time detection, can achieve fast and accurate identification of sheep behavior.

## Algorithm deployment and testing

In real breeding scenarios, the computing power of edge devices is often low. Therefore, in order to verify the practicality of the method in this paper, the YOLOv8n model and the PD-YOLOv8n model are deployed on the Rockchip RK3399Pro development board for experiments. The RK3399Pro development board supports mainstream frameworks such as PyTorch and TensorFlow, and has the characteristics of small size, low power consumption, and high computing performance. RK3399Pro also integrates an AI neural network processor NPU with a computing power of up to 3.0 Tops.

**Fig 15:**
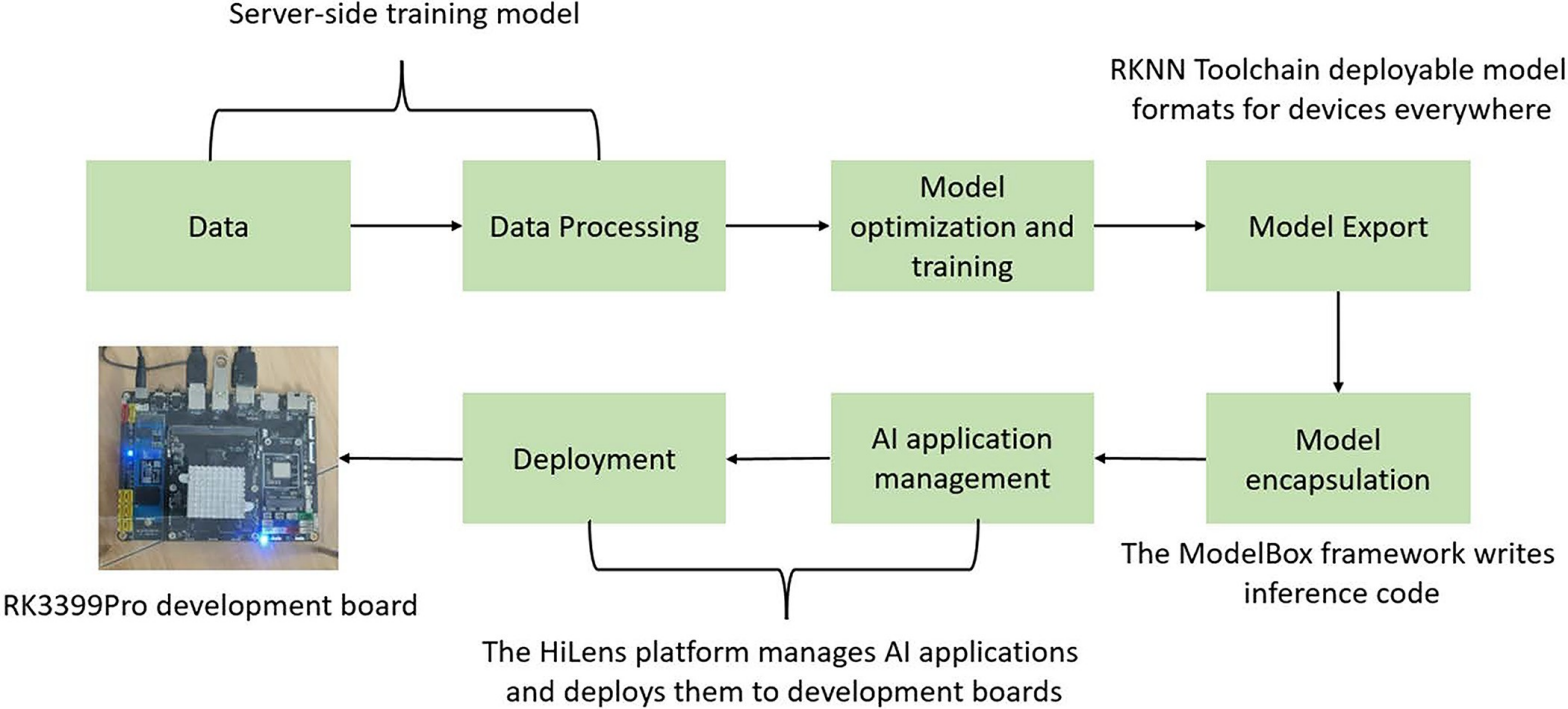
Model training and RK3399Pro platform deployment.

**Fig 16:**
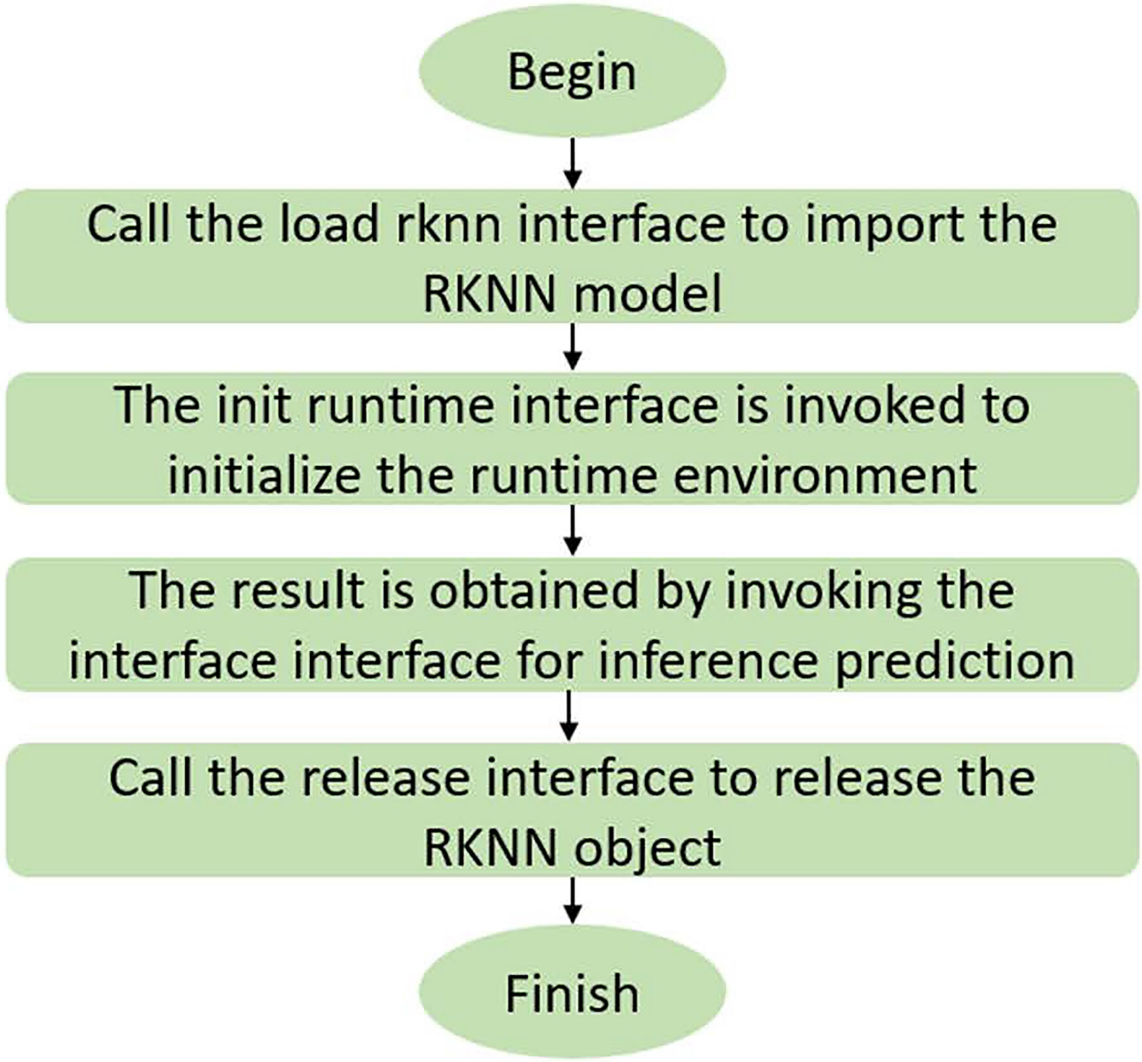
RK3399Pro platform inference flow chart.

**Fig 17:**
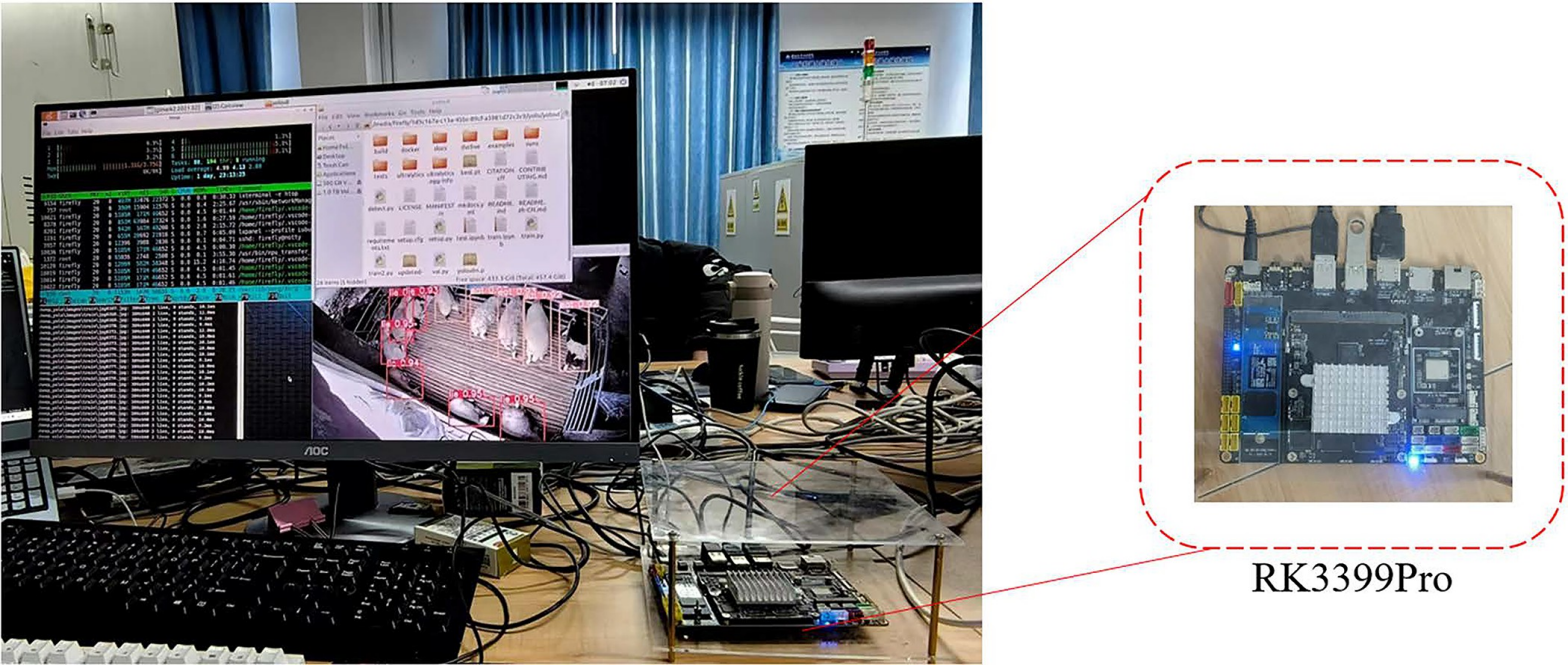
Algorithm deployment platform build.

During the model deployment process, we used the RKNN framework. First, the model trained using PyTorch on the PC side is converted to the ONNX format, and then the ONNX model is converted into an RKNN model suitable for the Rockchip platform through the RKNN toolchain provided by Rockchip. Subsequently, Huawei Cloud’s open source framework ModelBox is used to implement model loading and inference, so that the model can run on the Rockchip micro platform. The model training and deployment process on the RK3399Pro platform is shown in Fig 15. After the model is deployed, the inference process of the RK3399Pro platform is shown in Fig 16, and the display of the RK3399Pro platform is shown in Fig 17. Introducing Mean Average Precision (mAP) and inference speed (FPS) as evaluation metrics, the YOLOv8n model is compared with the PD-YOLO model.The comparison experiment results are shown in Table 5.

**Table 5:** FPS comparison of different models on RK3399Pro development board.

Model	mAP50 (%)	FPS(Frame/s)
*YOLOv8n*	*87*.*2*	*29*
*PD−YOLO*	*92*.*1*	*33*

It can be seen from Table 5 that in embedded devices, the PD-YOLOv8n model still performs well, with a detection speed of 33FPS, which is 13.7% higher than that of YOLOv8n model, and the average accuracy is 4.9 percentage points higher than that of YOLOv8n, which can meet the actual needs of real-time performance of sheep behavior recognition algorithm in industrial applications and embedded device deployment.

## Discussion

As with any study, our work has some limitations that need to be considered. One major potential factor affecting the accuracy of our model is that differences in camera location may affect the accuracy of the behavioral detection model. Secondly, in farm environments with a mix of large and small sheep, small sheep often hide under the body of large sheep, which may hinder the detection of sheep behavior. The next step is to explore multi-camera angle behavior detection for housed sheep in different scenarios, and our future work will focus on improving the robustness of the model to the environ-ment, as well as exploring new strategies to address these challenges.

## Conclusions

The focus of this study is to identify the daily behavior of sheep in a housing scenario. For this purpose, we propose a PD-YOLO model based on the YOLOv8 framework. First, to solve the severe occlusion problem caused by sheep herding, we propose the PCBAM module, which can utilize both spatial and channel information to reduce repetitive processing and loss of feature information. We embed the PCBAM module in the neck net-work to improve the processing capability of the features extracted from the backbone network. Then, to reduce the number of model parameters as well as the computational complexity, we replace the ordinary convolution in the backbone network with the DSConv convolution, which makes our model more usable in mobile devices and re-source-constrained environments. The experiments were conducted on a self-constructed daily behavioral dataset of sheep housed in pens with a high number of farmed sheep. The experimental results showed that PD-YOLO improved mAP by 3.3% over YOLOv8. It is worth noting that PD-YOLO improved sheep feeding behavior by 8.5% over YOLOv8, and reduced model size and FLOPs by 13.3% and 12.1%, respectively. In terms of detection speed, PD-YOLO improves by 3.2 FPS over YOLOv8.PD-YOLO has a higher mAP compared to Faster R-CNN, RTMDet, YOLOv3, YOLOv4s, YOLOv5n, YOLOv6n, YOLOv7-tiny, YOLOv9-T and YOLOv10n with mAPs of 12.5%, 2.9%, 8.9%, 2.6%, 5.3%, 9.1%, 6.3%, 7.7% and 8.4%. Taken together, our model has the best overall performance. Finally, we ported the PD-YOLO model to the RK3399Pro development board for experimentation, and the FPS was 33, which met the requirement of real-time detection, and further verified the feasibility of real-time detection of sheep behavior in real farming scenarios. However, there are still some shortcomings in our method, such as a single camera angle, which may limit the comprehensive observation of sheep behavior. In future studies, we will overcome this limitation and apply our model to sheep farms.
